# Supplementary material for: Identifying branch-specific positive selection throughout the regulatory genome using an appropriate proxy neutral
Source: BMC Genomics. 2020 May 13;21:359. doi: 10.1186/s12864-020-6752-4 (PMC7222330; doi:10.1186/s12864-020-6752-4)
Supplement: Supplementary file 1 — Additional file 1: Supplementary information. [file 12864_2020_6752_MOESM1_ESM.docx]

**SUPPLEMENTARY INFORMATION**

**Supplementary table 1**

| **Locus** | **Nearest Gene** | **Distance from nearest gene** | **Function of the nearest gene** |
| --- | --- | --- | --- |
| chr2:236773664-236774209 | AGAP1 | 370.9 kb | GTP binding and phospholipid binding and endocytosis |
| chr4:178272899-178273376 | NEIL3 | 41.9 kb | Nucleic acid binding and single-stranded DNA binding and telomere C-strand synthesis. |
| chr9:16717947-16718304 | BNC2 | 307.7 kb | Zinc finger protein functioning in skin color saturation and associated with idiopathic scoliosis. |
| chr16:82295069-82295368 | MPHOSPH6 | 91.5 kb | Deadenylation-dependent mRNA decay and rRNA processing in the nucleus and cytosol. |

**Supplementary Table 2**

| Dataset | Direct link |
| --- | --- |
| 100 vertebrate alignment | <http://hgdownload.soe.ucsc.edu/goldenPath/hg19/multiz100way/> |
| NCBI RefSeq Human Known genes | http://hgdownload.soe.ucsc.edu/goldenPath/hg19/database/ncbiRefSeqCurated.txt.gz |
| UCSC Human Known genes | http://hgdownload.soe.ucsc.edu/goldenPath/hg19/database/knownToRefSeq.txt.gz |
| long non-coding RNAs | http://hgdownload.soe.ucsc.edu/goldenPath/hg19/database/lincRNAsTranscripts.txt.gz |
| Human tRNA | http://hgdownload.soe.ucsc.edu/goldenPath/hg19/database/tRNAs.txt.gz |
| Human microRNA and snoRNA | https://github.com/wodanaz/adaptiPhy/blob/master/wgRNA.bed |
| Total human mRNA | http://hgdownload.soe.ucsc.edu/goldenPath/hg19/database/all_mrna.txt.gz |
| Synthesis of all human open chromatin, DNAseI-seq, FAIRE-seq, ChIP-seq | http://genome.ucsc.edu/cgi-bin/hgFileUi?db=hg19&g=wgEncodeOpenChromDnase |
| Thurman et al, 2012. 125 different cell types and tissues | http://hgdownload.soe.ucsc.edu/goldenPath/hg19/database/wgEncodeAwgDnaseMasterSites.txt.gz |
| Curated list of promoters and enhancers from HoneyBadger / | <https://personal.broadinstitute.org/meuleman/reg2map/HoneyBadger_release/prom/clustering_1kb/BED_files/>  <https://personal.broadinstitute.org/meuleman/reg2map/HoneyBadger_release/enh/clustering_10kb/BED_files/>  https://personal.broadinstitute.org/meuleman/reg2map/HoneyBadger_release/dyadic/clustering_1kb/BED_files/ |
| Human Vista Enhancers | http://hgdownload.soe.ucsc.edu/goldenPath/hg19/database/vistaEnhancers.txt.gz |
| CpGislands | http://hgdownload.soe.ucsc.edu/goldenPath/hg19/database/cpgIslandExt.txt.gz |
| Microsatellite repeats | http://hgdownload.soe.ucsc.edu/goldenPath/hg19/database/microsat.txt.gz |
| Interrupted Rpts - Fragments of Interrupted Repeats Joined by RepeatMasker ID | http://hgdownload.soe.ucsc.edu/goldenPath/hg19/database/nestedRepeats.txt.gz |
| Human NumtS mitochondrial sequence | http://hgdownload.soe.ucsc.edu/goldenPath/hg19/database/numtS.txt.gz |
| Simple Tandem Repeats by TRF | http://hgdownload.soe.ucsc.edu/goldenPath/hg19/database/simpleRepeat.txt.gz |


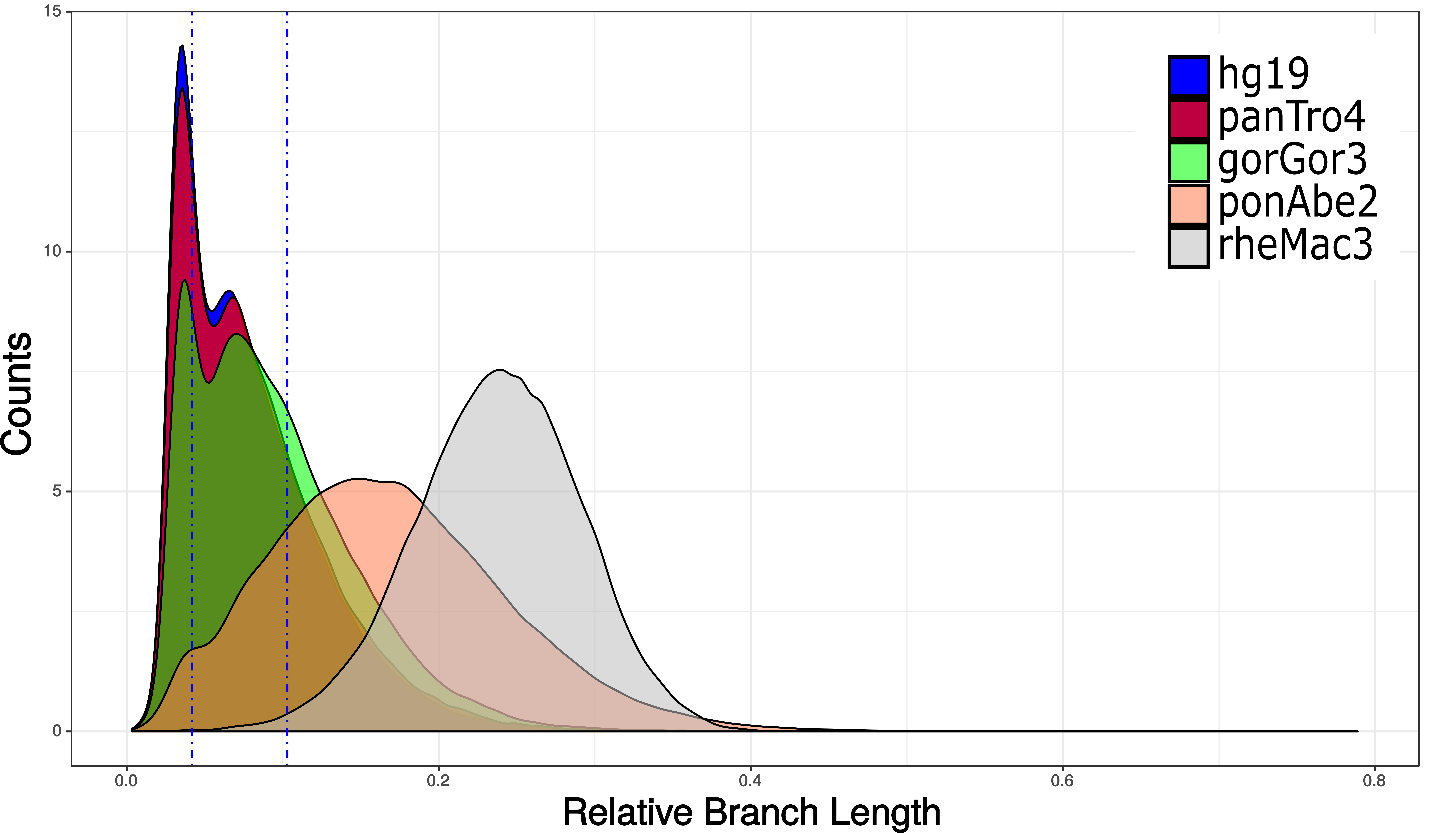


**Figure S1:** Distribution of relative branch length across non-functional and putatively neutrally evolving elements.

**
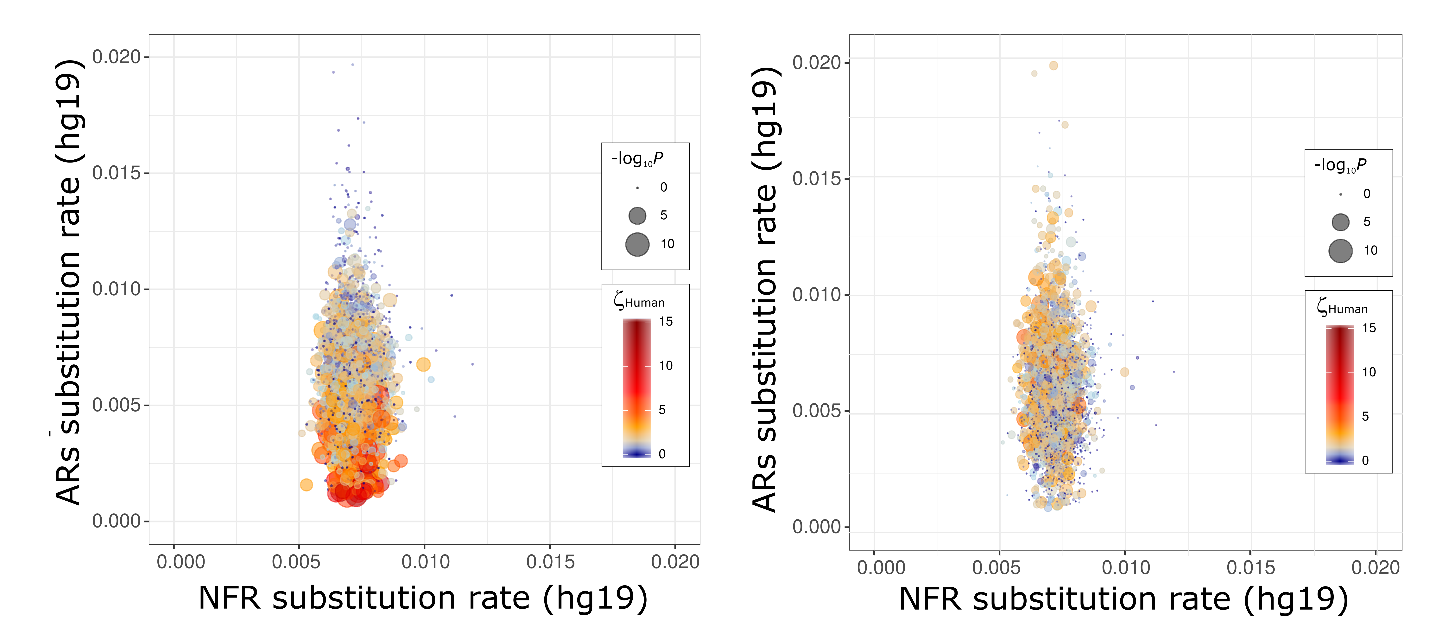
**

**Figure S2. AR elements bias the estimation of positive selection.** The distribution of substitution rates among ARs (y-axis) used as a reference is appreciably wider than in post-filtered non-functional regions (x-axis). This increases the rate of false positives of query regions using reference regions that are more conserved. Color gradient depicts the magnitude of ζ while size depicts the significance (-Log_10_ *P*value) of a DHS query. Cold colors depicts regions that are neutral or constrained, while warmer colors depict more hits of positive selection. Tests using local AR elements (left), tests using global NFR elements in the reference (right).

**
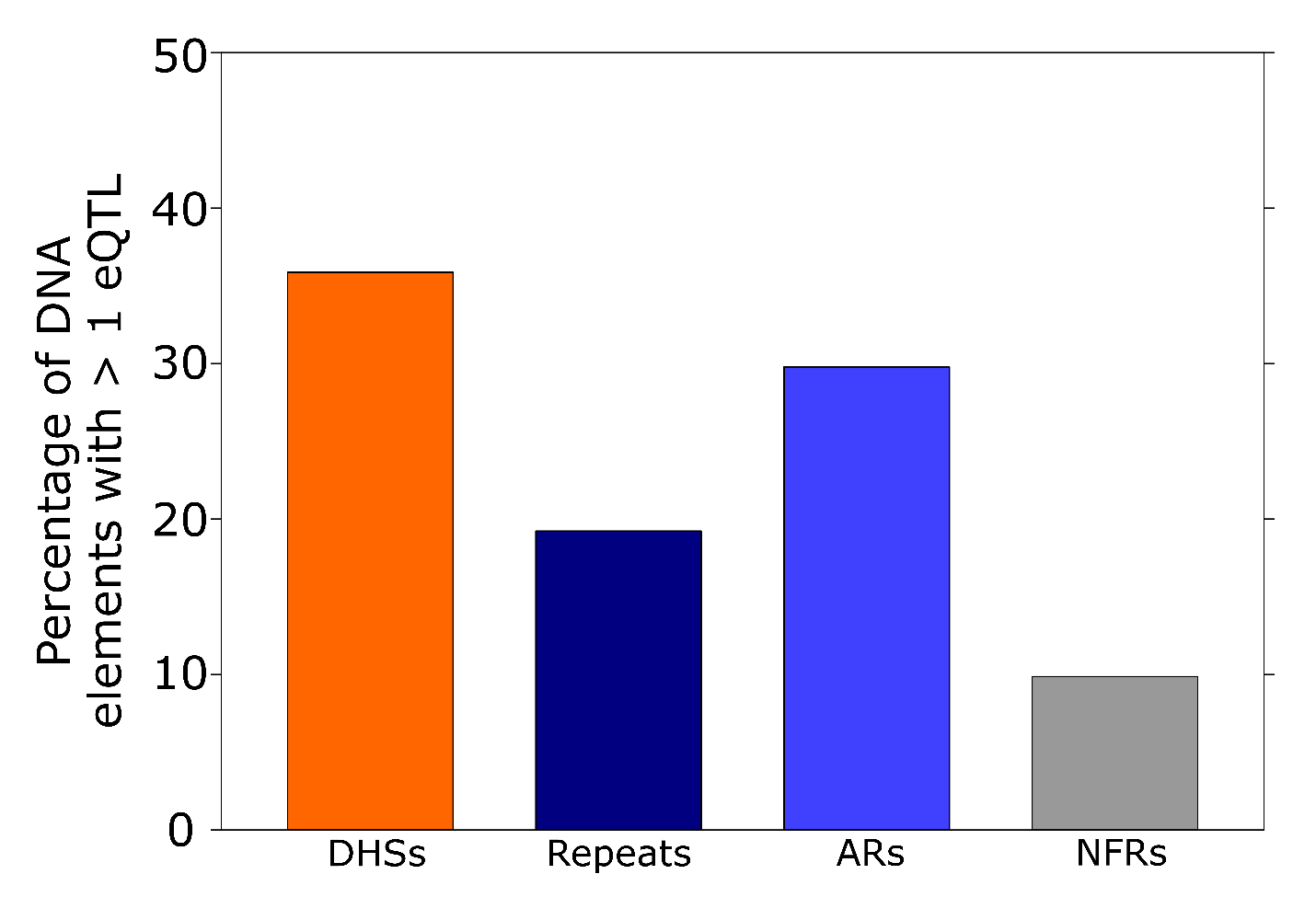
**

**Figure S3. Percentage of DNA elements with at least one multi-tissue eQTL.** Many repeats and ancestral repeats contain at least 1 multitissue eQTL. Almost 9% of our random list of 5104 non-functional elements contain at least 1 eQTL.

**
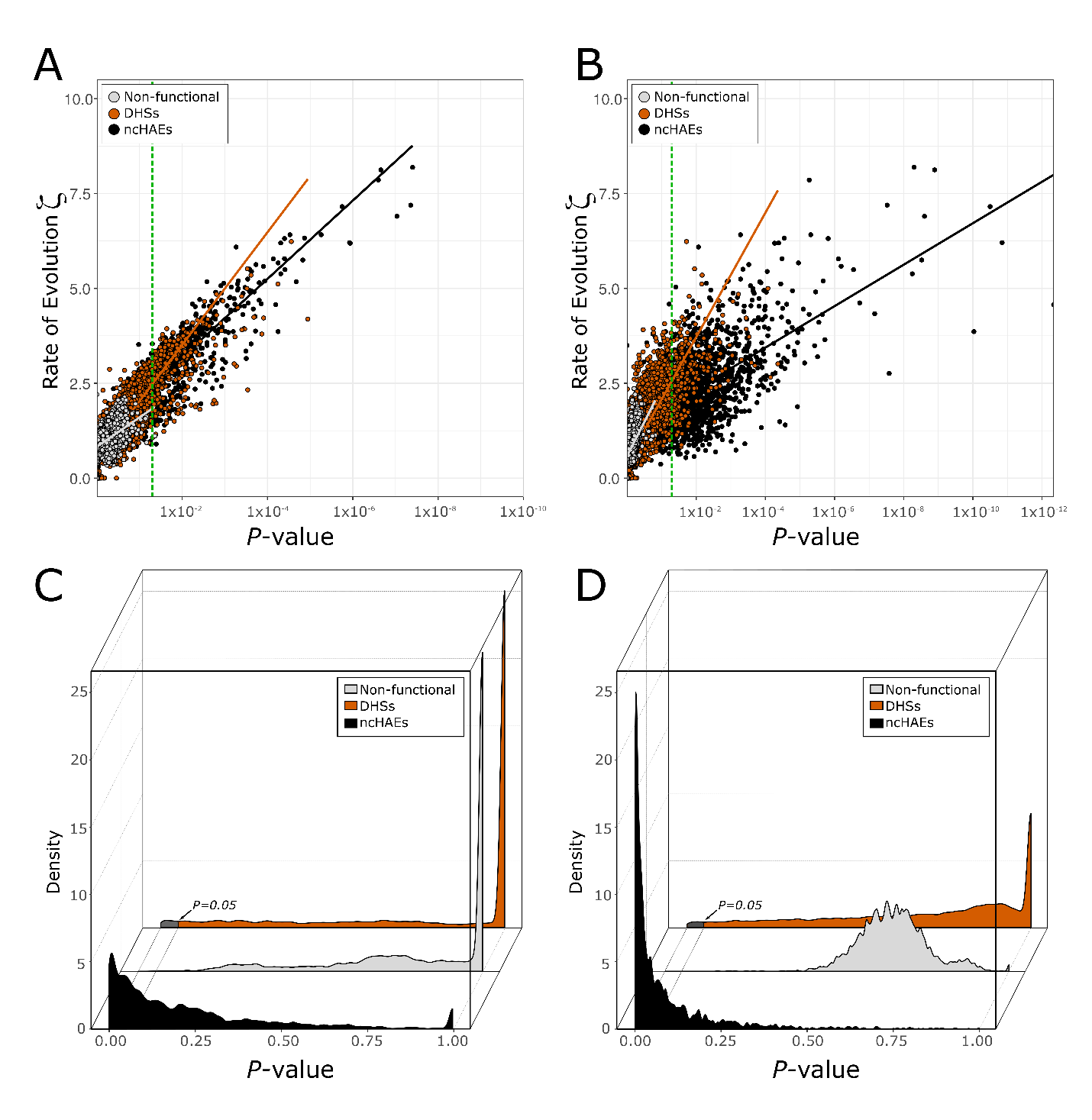
**

**Figure S4: Correlation between evolutionary ratio and *P*-value.** Rate of evolution in the human branch using global neutral proxy for DHSs (orange), non-functional elements (gray), and noncoding ncHAEs (black). The green dotted line depicts significance at the 0.05 level. **A.** All the Spearman correlation coefficients for our framework using *HyPhy* are strongly significant (*P* < 2.2 x10^-16^), and very high (rho = 0.86 for ncHAEs and rho = 0.80 for DHSs) except for non-functional data (rho = 0.60). **B.** All the Spearman correlation coefficients for *phyloP* scores of acceleration using our global neutral proxy are also strongly significant (*P* < 2.2 x10^-16^), and very high (rho = 0.55 for ncHAEs and rho = 0.90 for DHSs) except for non-functional data (rho = 0.47). **C-D.** Density distributions of *P*-value among different classes of DNA elements including non-functional sequences (gray); a set of 11649 DHSs (orange) from Thurman et al (2012); and the distribution of *P*-values provided by our tests for ncHAEs (black) for *adaptiPhy* **(C)** and *phyloP* scores of acceleration **(D)**. Significant distributions of elements scoring high for positive selection have been highlighted.


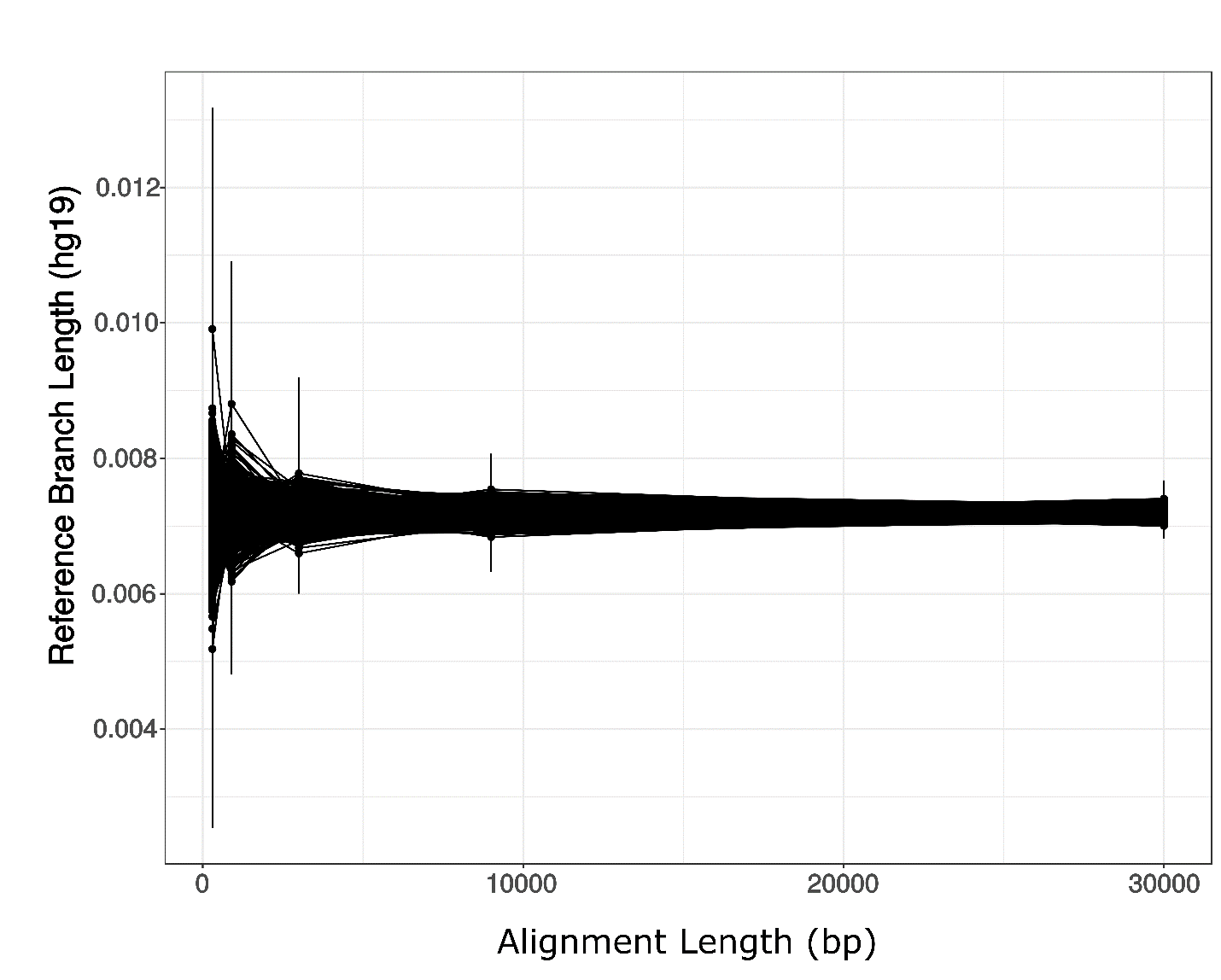


**Figure S5. Effect of alignment length on reference branch length.** Variation of branch length in the human branch relative to the alignment length among concatenated references from real non-coding data (black).


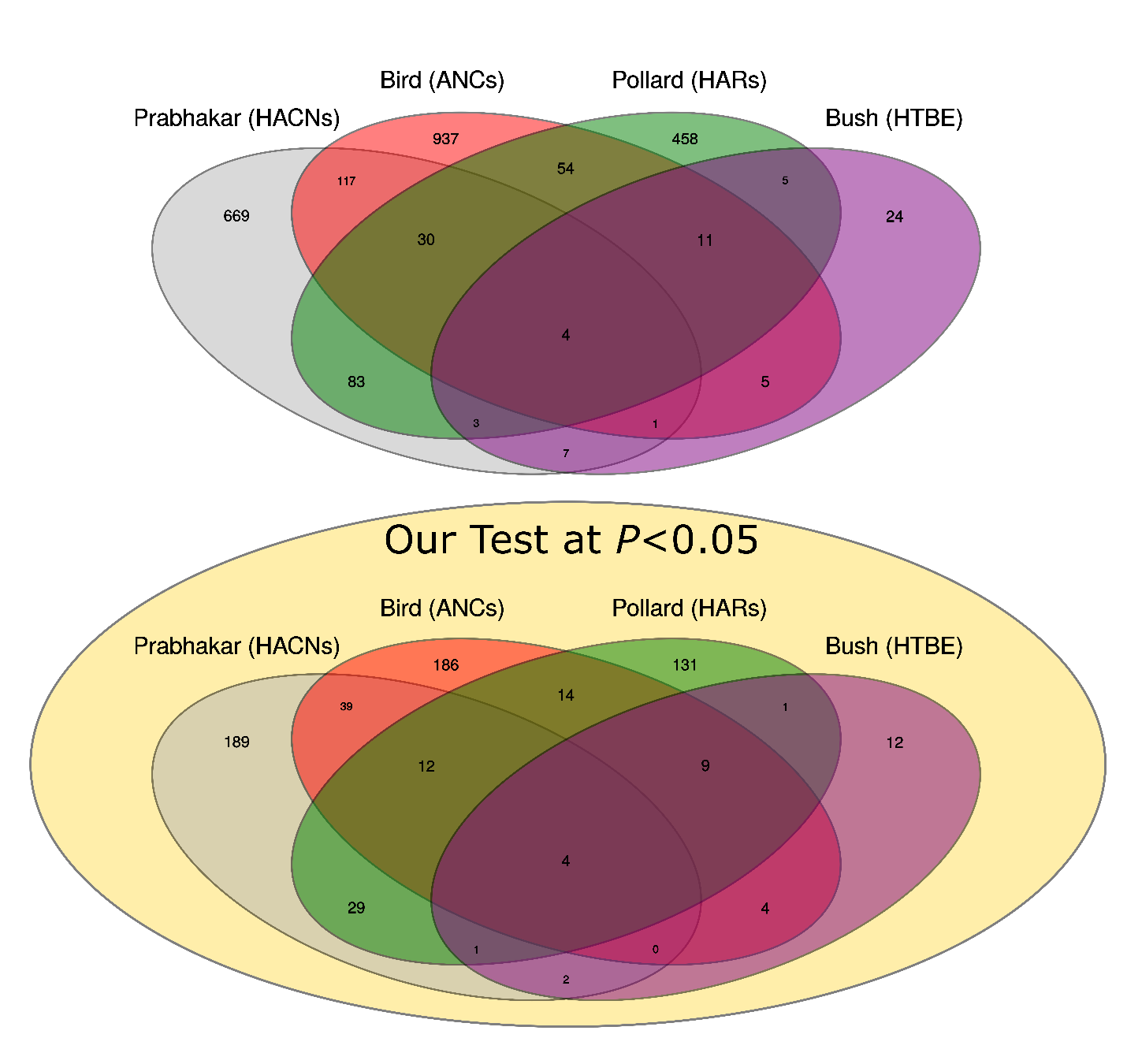


**Figure S6: Overlaps among different datasets of human accelerated elements.** **A.** Total overlap between HACNs, ANCs, HARs and HTBEs (Pollard, Salama, King, et al. 2006; Bird et al. 2007; Bush and Lahn 2008; Prabhakar et al. 2008). **B.** Overlap among all human accelerated elements in A that scored high for positive selection with *adaptiPhy* at a *P* < 0.05.


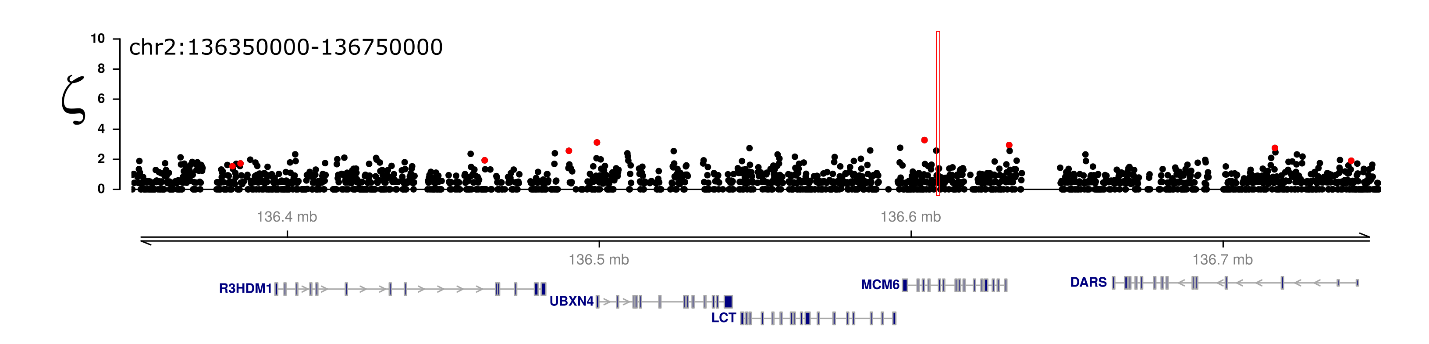


**Figure S7:** **Sliding windows analysis of the rate of evolution along one of the best known human sweeps.** Distribution of the rate of evolution as ζ along 400 kb around the LCT locus. This locus contains several polymorphisms that are associated with the evolution of lactase persistence. The location of these polymorphisms is highlighted in pink, and the red dots represent windows of 300 bp where ζ scored significant for positive selection (P < 0.05).
